# Stress and pluripotency

**DOI:** 10.3389/fcell.2014.00032

**Published:** 2014-08-06

**Authors:** Bor L. Tang

**Affiliations:** ^1^Department of Biochemistry, Yong Loo Lin School of Medicine National University of SingaporeSingapore; ^2^NUS Graduate School for Integrative Sciences and Engineering National University of SingaporeSingapore

**Keywords:** pluripotency, reprogramming, stress, physiological, stem cells, Oct4 expression

Vacanti and colleagues recently reported the generation of mouse cells exhibiting pluri/totipotency by stress treatment, and showed that these cells can give rise to chimeras and clonal adults (Obokata et al., 2014a,b). The findings were controversial, and the authors have now retracted the papers, citing multiple critical errors within the reports that impaired the credibility of the findings as a whole. The central notion of this stimulus-triggered acquisition of pluripotency (STAP), and how the idea might have evolved from other notions, is nonetheless worth a moment of thought. In view of the fact that generation of STAP cells beyond the authors' labs have not yet been definitely reported thus far (http://www.ipscell.com/stap-new-data/), one could perhaps surmise that the STAP process, even if real, is difficult to reproduce. The big question though, is whether generation of pluripotent cells by stress alone is at all plausible.

How could stress alone result in induced pluripotency, indicated at the first instance by the formation of Oct4 expressing colonies? Regardless of the type of stress, and the steps involved, reprogramming is causally related to and driven by (not merely accompanying) expression of reprogramming genes such as Oct4, Sox2, and Nanog. In this regard, it is notable that stress-activated forkhead boxO 1 (FoxO1) has binding sites on the promoters of both Oct4 and Nanog in embryonic stem cells (ESC), and FoxO1 is known to be essential for maintenance of pluripotency of ESC (Zhang et al., 2011). Stress could also potentially reduce the activity of the nucleosome remodeling and deacetylation (NuRD) repressor complex (Opsahl et al., 2010) which acts in attenuating the downstream targets the reprogramming factors (Luo et al., 2013) thus promoting efficiency of reprogramming. A variety of stresses activate autophagy (Pietrocola et al., 2013), which has been shown to be an important early step for somatic cells to be reprogrammed into induced pluripotent stem cells (Wang et al., 2013).

Although these connections exist, it is unclear if the expression of any of the factors above indeed occurs with the procedure used, and could be sustained above threshold levels required to drive reprogramming in somatic cells. The above view, which suggestively equates the mechanism of stress induced reprogramming to that mediated by the Yamanaka factors, is also oversimplified. Reprogramming goes through multiple phases, with the initial phase being stochastic, and not deterministic of eventual acquisition of pluripotency. Expression of pluripotency genes such as Oct4 and Sox2 may well be elevated in a good number of cells in a transfected population, but that does not necessarily culminate in successful reprogramming of every such cell (Buganim et al., 2012).

On the other hand, stress also activates major barriers against reprogramming, the most prominent being p53 and its downstream activities (Krizhanovsky and Lowe, 2009). Acidosis, which will accompany the primary method of low pH incubation in STAP cell generation, activates p53 (Dregoesc and Rainbow, 2009; Lamonte et al., 2013). Other than causing massive death in the culture, which is a phenomenon reported by most if not all attempts to reproduce STAP cells elsewhere, p53 activation would also suppress Nanog expression (Lin et al., 2005; Cinghu et al., 2014). Indeed, it would be difficult to envisage how reprogramming, even infrequently, could predominate over senescence and apoptosis induced by p53 activation alone. This is unless if one starts with p53-deficient cells, or for some reason, p53 defective cells are selected in the process. The genome of cells with defective p53 will however, not likely to be readily germ line transmissible.

It is also worth noting that the association of stress with pluripotency has been made previously by Obokata and colleagues, as well as others. The authors had previously reported the isolation of cytosphere-forming multi/pluripotent stem cells from several adult tissues capable of differentiating into all three germ layers using “vigorous” trituration (Obokata et al., 2011). Trituration was subsequently suggested and recommended as a method in conjunction with low pH treatment for the production of STAP cells. It was unclear though if the trituration merely served to disperse cell clumps, or had it contributed toward pluripotency induction in the authors' earlier paper. Another report had also indicated that pluripotent stem cells known as Multilineage differentiating stress-enduring (Muse) Cells, could be isolated from adipose tissues using severe cellular stress conditions (Heneidi et al., 2013). The notion of the existence of pluripotent stem cells in multiple adult tissues have been expounded by several groups (Jiang et al., 2002; Brons et al., 2007; Ratajczak et al., 2014), but this notion has remained controversial (Danova-Alt et al., 2012; Miyanishi et al., 2013; Szade et al., 2013). It is, however, conceivable that the idea of STAP may have “evolved” from the perception that stress-related protocols have aided the isolation of adult stem cells.

There are of course major differences between the isolation of pluripotent stem cells from adult tissues compared to *de novo* induction of pluripotency from lineage committed somatic cells. Quiescent adult stem cells in some niches could indeed be induced to proliferate by injury or stress (Wilson et al., 2008; Rodgers et al., 2014). To think that induced pluripotency of fully differentiated cells could be achieved by stress alone would be quite a massive leap of faith. Nature, however, has a habit of surprising us. With the papers officially retracted and the major findings now deemed untenable, one might yet find investigations into possible connections between stress-induced signaling and reprogramming yielding interesting unknowns.

## Conflict of interest statement

The author declares that the research was conducted in the absence of any commercial or financial relationships that could be construed as a potential conflict of interest.
